# Crystallographic Orientation Influence on the Serrated Yielding Behavior of a Single-Crystal Superalloy

**DOI:** 10.3390/ma6020437

**Published:** 2013-01-31

**Authors:** Mikael Segersäll, Johan J. Moverare

**Affiliations:** 1 Department of Management and Engineering, Linköping University, Linköping 58183, Sweden; E-Mail: johan.moverare@liu.se; 2 Siemens Industrial Turbomachinery AB, Finspång 61283, Sweden

**Keywords:** single-crystals, superalloy, yield phenomena, tension/compression asymmetry, dynamic strain aging

## Abstract

Since Ni-based single-crystal superalloys are anisotropic materials, their behavior in different crystal orientations is of great interest. In this study, the yielding behavior in both tension and compression for 〈001〉, 〈011〉 and 〈111〉 oriented materials at 500 °C has been investigated. The 〈011〉 direction showed a serrated yielding behavior, a great tension/compression asymmetry in yield strength and visible deformation bands. However, the 〈001〉 and 〈111〉 directions showed a more homogeneous yielding, less tension/compression asymmetry in yield strength and no deformation bands. Microstructure investigations showed that the serrated yielding behavior of the 〈011〉 direction can be attributed to the appearance of dynamic strain aging (DSA) and that only one slip system is active in this direction during plastic deformation.

## 1. Introduction

Ni-based single-crystal superalloys are used as blade material in gas turbines and aero engines thanks to their excellent resistance to mechanical and chemical degradation at elevated temperatures [1,2]. By using single-crystal instead of poly-crystal material in gas turbine blades, both fatigue and creep properties are enhanced. Commonly, the 〈001〉 direction is aligned along the length of the turbine blades, since this direction has the lowest Young’s modulus of the 〈001〉, 〈011〉 and 〈111〉 directions, and therefore best fatigue properties. However, the other main directions, 〈011〉 and 〈111〉, are also of great interest since crack propagation in turbine blades sometimes is dependent on the properties in these directions.

It is well recognized that Ni-based single-crystal superalloys exhibit a number of surprising mechanical properties, including an increase in yield strength with temperature, strong orientation dependence of the yield stress and a tension/compression asymmetry [3,4,5,6]. Traditionally the dependence of the flow stress on both temperature and crystallographic orientation has been attributed to the cross-slip of dislocations from the octahedral planes {111}, to the cube planes {100}, in the γ'-precipitates [7,8]. However, according to Lall *et al.* [9], the cross-slip is not only aided by the resolved shear stress on the {100} cross-slip plane, but also by the stress tending to constrict the a/6〈112〉 Shockley partial dislocations on the primary glide plane, often referred to as “the core width effect”. This is believed to be the main reason for the tension/compression asymmetry at low and intermediate temperatures. Later on, more advanced models were derived assuming that deformation after cross-slip can occur by the lateral motion of superkink segments, lying on the {111} planes, which links the Kear-Wilsdorf locks that were formed on the {010} planes during cross-slip [10,11].

Sometimes also a serrated yielding behavior is in progress during plastic deformation. Many metallic materials show a serrated flow, also known as the Portevin-Le Chatelier (PLC) effect. The reason for this behavior is the interaction between diffusing solute atoms and moving dislocations, more commonly known as dynamic strain aging (DSA). It is well known that the DSA phenomena may lead to a serrated yielding effect and in the literature one can find that DSA occur in superalloys at temperatures from 260 to 800 °C [12]. Research has showed that also interaction of planar slip bands can cause serrated yielding behavior [13]. Other literature [14] reported a plastic deformation inhomogeneity in different crystallographic directions for the DD8 single-crystal superalloy. In compression at 550 °C, the 〈110〉 direction showed a stronger inhomogeneity compared to 〈001〉 and 〈111〉. The inhomogeneity for the 〈110〉 direction was explained by dislocation shearing of γ'-particles, while for the most stable directions, 〈001〉 and 〈111〉, the deformation process rather was controlled by Orowan by-passing. Miner *et al.* [15] reported a serrated yielding behavior followed by loud pops for the 〈011〉 direction when loading in tension at 760 °C. This was explained by the fact that only the primary octahedral slip system,$\left(111\right)\left[\bar{1}01\right]$, was active for the 〈011〉 direction while several octahedral slip systems were active for the 〈001〉 direction.

Studies concerning the behavior of 〈001〉 oriented single-crystal superalloys are quite commonly found in the literature. However, comparisons of behavior between different crystal orientations are more rare. The purpose of this paper is therefore to investigate the differences in intermediate temperature yielding behavior, both in tension and compression, between the three crystallographic directions, 〈001〉, 〈011〉 and 〈111〉.

## 2. Experimental Procedure 

In this study the Ni-based single-crystal superalloy MD2 with chemical composition Ni, 5.1 Co, 6.0 Ta, 8.0 Cr, 8.1 W, 5.0 Al, 1.3 Ti, 2.1 Mo, 0.1 Hf and 0.1 Si was investigated. The material was solution heat treated at 1275 °C for 8 h followed by a two-stage aging process with 3 h at 1100 °C and 24 h at 850 °C. Test specimens were machined from cast bars and the deviation from the ideal orientation was less than 2° for the 〈011〉 and 〈111〉 specimens, but varied between 6° and 12° for the 〈001〉 specimens. The stress–strain response during monotonic tensile and compressive loading was investigated using a servohydraulic testing machine. The tests were conducted at 500 °C under displacement control, while monitoring strain and load, with an initial strain rate of ~10^−5^ s^−1^. The samples were carefully aligned to prevent buckling. The tests were interrupted when approximately 1% plastic strain was achieved, and the specimens were then unloaded, cooled rapidly to room temperature (RT) and removed from the test machine. After the tests, all specimens were investigated by stereomicroscopy before they were cut parallel to the loading direction for further investigation by scanning electron microscopy (SEM) in a Hitachi SU70 SEM. The SEM samples were prepared by grinding and mechanical polishing, but no samples were etched. Acceleration voltages from 10 to 20 kV were used during the SEM investigation. Most images were taken using backscattered electrons and channelling contrast. Electron backscatter diffraction (EBSD) analysis was performed using an Oxford Instrument Nordlys detector.

## 3. Results and Discussion

### 3.1. Mechanical Testing 

**Figure 1:**
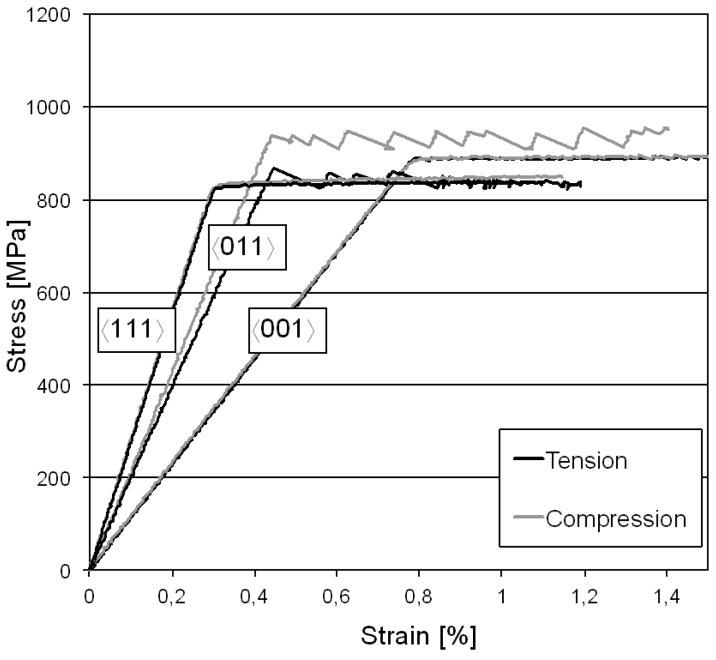
Tension and compression tests at 500 °C for 〈001〉, 〈011〉 and 〈111〉 directions.

The results from tensile and compressive tests at 500 °C for the three crystallographic directions 〈001〉, 〈011〉 and 〈111〉 are presented in Figure 1.

As expected, the 〈111〉 direction showed the highest stiffness of all directions, approximately 300 GPa in both tension and compression. The 〈011〉 direction showed a higher stiffness in compression compared to tension, approximately 220 GPa compared to 210 GPa. This difference can be attributed to the deviations from the ideal crystal orientation connected to each specimen. The 〈001〉 direction showed the lowest stiffness, approximately 115 GPa. Single-crystal superalloys exhibit a yield strength tension/compression asymmetry and in this study greatest asymmetry was found for the 〈011〉 direction, where the compressive yield strength was around 70 MPa higher compared to that in tension. However, the 〈001〉 and 〈111〉 directions instead showed similar yield strengths in tension and compression at this temperature, *i.e.*, no significant asymmetry was found for these directions. In addition to the tension/compression asymmetry, the 〈011〉 direction also showed a significant serrated yielding behavior, both in tension and compression. On the other hand, yielding for the 〈001〉 and 〈111〉 directions was more homogeneous. The highest yield strength was found for the 〈011〉 direction in compression, 940 MPa, followed by the 〈001〉 direction where the yield strengths in tension and compression were approximately 880 MPa. In tension, the 〈011〉 direction showed a yield strength of 865 MPa. The 〈111〉 orientation was the weakest of all directions; here the yield strength was approximately 825 MPa in both tension and compression.

**Figure 2:**
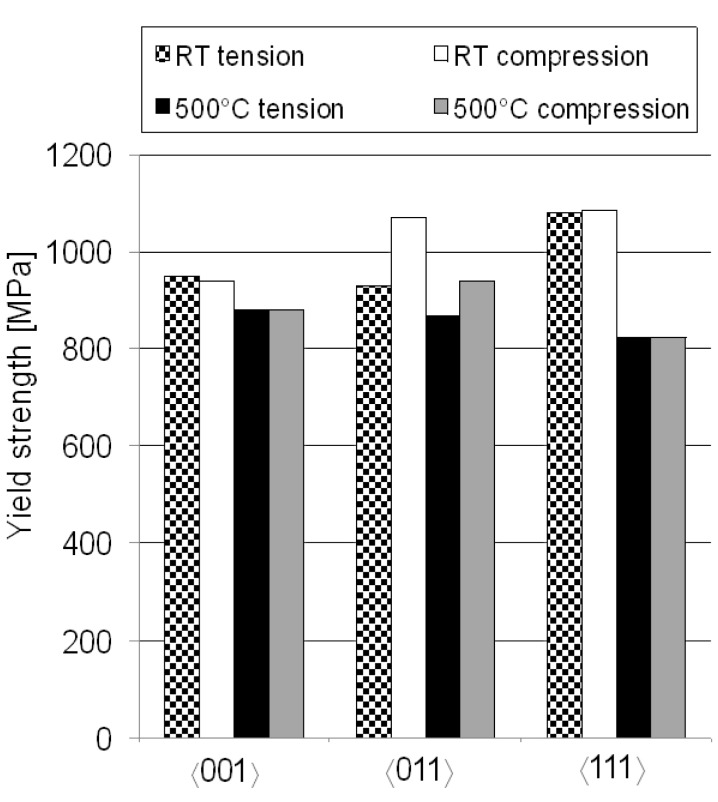
Yield strengths for 〈001〉, 〈011〉 and 〈111〉 directions at RT and 500 °C. RT yield strengths are taken from [5].

The results can be compared to another study [5] where the same tests were conducted, with the same alloy, at RT instead of 500 °C. In Figure 2 the yield strengths for all directions at both temperatures are summarized in one graph.

At both temperatures, the greatest tension/compression asymmetry in yield strength was found for the 〈011〉 direction. However, at RT no serrated yielding behavior was found for the 〈011〉 direction. This indicates that serrated yielding depends on diffusion and DSA. Further comparison to that study shows that the 〈111〉 direction had similar or even higher yield strength compared to the other directions at RT but at 500 °C this direction is the weakest. The 〈111〉 direction therefore show the biggest reduction in yield strength, approximately 25% when going from RT to 500 °C. For the 〈001〉 direction the same comparison shows a reduction in yield strength of around 7% only. This assumes that the yield strengths in tension and compression are approximately the same for those directions. When looking at the 〈011〉 direction, where the greatest tension/compression asymmetry was found, the tensile yield strength is 7% lower at 500 °C compared to RT while the compressive yield strength decreases 12% when going from RT to 500 °C.

### 3.2. Microscopy 

**Figure 3:**
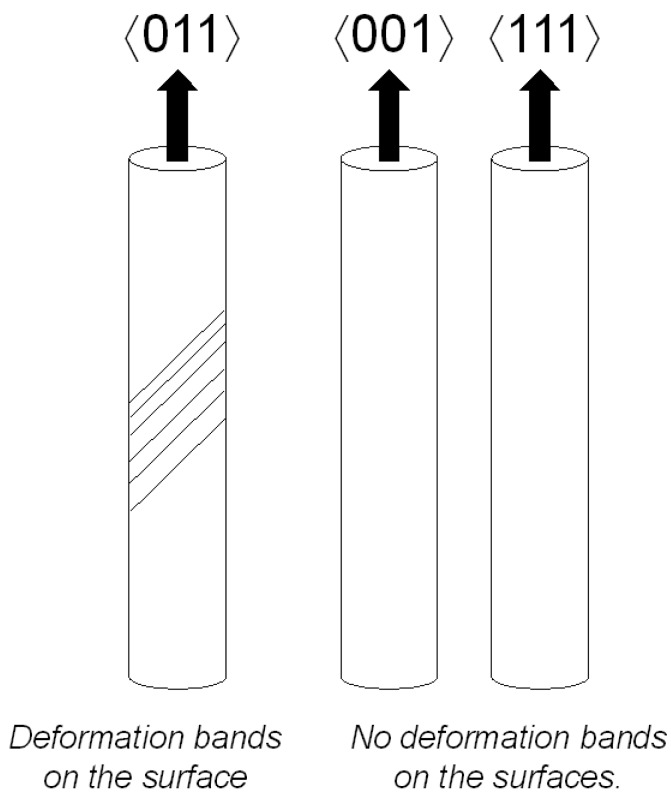
A sketch of the deformation band appearance on the specimen surfaces.

Specimen investigation by stereomicroscopy showed clear crystallographic deformation bands stretching over the 〈011〉 specimen surfaces, both in tension and compression. However, for 〈001〉 and 〈111〉 oriented specimens no clear deformation bands were visible on the surfaces. Unfortunately, since no specimens were polished prior to testing, it was very difficult to obtain high quality images of the deformation bands appearing on the 〈011〉 oriented specimen surfaces, and therefore only a sketch showing the deformation band appearance is shown in Figure 3. The slip traces on the 〈011〉 oriented specimens are consistent with slip along the $\left(111\right)\left[\bar{1}01\right]$ slip system.

**Figure 4:**
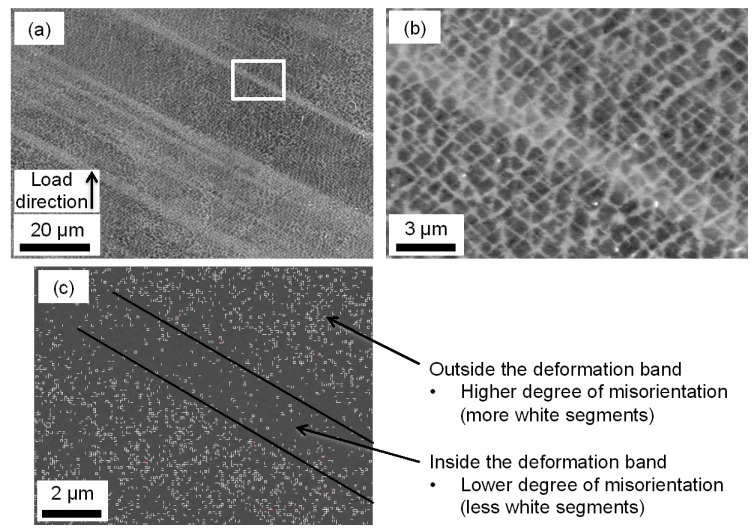
The 〈011〉 specimen subjected to compressive stress; (**a**) a backscattered electron image of deformation bands at 1200 times magnification; (**b**) same deformation band as in (**a**) at 7000 times magnification; (**c**) EBSD map of such a deformation band, where each white segment corresponds to an orientation difference of 0.5°–1°.

Further investigation by SEM, using backscattered electrons, also showed clear deformation bands in the microstructure for the 〈011〉 specimens. Figure 4a shows the typical appearance of deformation bands in the γ/γ'-microstructure at low magnification for the 〈011〉 oriented specimen subjected to compressive loads. Investigations of the same deformation band at a higher magnification did not reveal any significant shearing of the γ/γ'-microstructure, Figure 4b. This is probably due to the fact that the tests were interrupted after 1% plastic deformation. Further deformation probably would have revealed dislocations cutting of the γ'-phase.

EBSD was performed to further examine the orientation variations between the deformation bands and the less deformed γ/γ'-microstructure. The mapping showed very small orientation variations, up to 1°, between the deformation bands and the less deformed microstructure. This means that twinning of the microstructure is not present within the deformation bands. In Figure 4c an EBSD map of such a deformation band is shown where the white segments indicate orientation differences from 0.5° to 1°. Interestingly, it is clear that the orientation differences (see white segements in Figure 4c) are concentrated outside the deformation band, while the band itself contains much less orientation differences. The orientation differences outside the band are very likely coupled to the misorientation between the γ- and γ'-phases. Inside the band, the γ/γ'-misorientation has probably been equalised by dislocations cutting the γ'-phase, resulting in less orientation differences. The deformation bands are therefore very likely bundles of glide bands, *i.e.*, dislocations that propagate through the crystal. Hence, the dislocation density within the deformation bands is higher compared to the less deformed microstructure. Deformation bands in 〈011〉 oriented material has been reported before in the literature [16], and in that study the deformation bands were detected as changes in γ-channel size within the microstructure. This seems not to be the case in this study.

Thus, the 〈011〉 direction shows deformation bands, which are bundles of glide bands, and a serrated yielding behavior while the other directions show stable yielding and no deformation bands. A serrated yielding behavior is often explained by DSA and this has very likely occurred within the glide bands found in the 〈011〉 oriented material. DSA is a phenomenon where moving dislocations are pinned by diffusing solute atoms and this is it more probable to occur in regions with a high dislocation density, *i.e.*, the deformation bands. This can explain the serrated yielding behavior showed by the 〈011〉 direction. The 〈001〉 and 〈111〉 directions showed no serrated yielding behavior and neither any glide bands in the microstructure. With no glide bands, the potential for DSA to occur is smaller compared to when glide bands are present. Once again comparing to the previous study [5], no serrated yielding effect was found in any direction when testing at RT. This is reasonable since DSA is a diffusion dependent process, and the interaction between solute atoms and moving dislocations are more likely to occur at 500 °C compared to RT.

Hence, DSA in combination with highly localized deformation bands is one reason for the serrated yielding behavior of the 〈011〉 direction. This is in line with previous research by Miner *et al.* [14], who concluded that only one slip system is active during yielding for the 〈011〉 direction while multiple slip systems are active in the other directions. Due to symmetry reasons, the 〈011〉 direction has 4 slip systems with the same Schmid factor, while the 〈001〉 direction has 8 and the 〈111〉 direction has 6 equivalent slip systems. However, since each specimen is not aligned exactly in the 〈011〉 direction, there will be one slip system that have a higher resolved shear stress and will operate as the primary slip system. Due to lower symmetry (less equivalent slip systems) of the 〈011〉 direction compared to the 〈001〉 and 〈111〉 directions, the 〈011〉 direction has a higher tendancy for single slip compared to the other orientations. When multiple slip systems are active, there will always be one system where dislocation glide can occur during plastic deformation and the yielding becomes more stable. However, when only one slip system is active, the dislocations in the glide bands can only propagate in one direction and are therefore sometimes stopped, if the stress is not big enough, before they can propagate any further. This leads to the serrated yielding effect. With only one slip system active, it is also more likely to obtain those concentrated glide bands which end up as topographic differences on the specimen surface. For the 〈001〉 and 〈111〉 directions, when multiple slip systems were active at the same time the surface becomes more homogenous with less topographic difference.

## 4. Conclusions 

To conclude this study, it is very clear that the 〈011〉 direction demonstrated a tension/compression asymmetry and a serrated yielding behavior in both tension and compression, at 500 °C. For this direction, deformation bands were also found both on the surface and in the γ/γ'-microstructure, and the deformation bands are very likely bundles of glide bands. The main reason for the serrated yielding behavior of the the 〈011〉 direction is the occurrence of DSA in combination with highly localized deformation bands. In contrast to this, the 〈001〉 and 〈111〉 directions showed a more homogeneous yielding behavior, no distinct deformation bands and no tension/compression asymmetry.
